# Mesencephalic Astrocyte-Derived Neurotrophic Factor (MANF) Protects Against Neuronal Apoptosis via Activation of Akt/MDM2/p53 Signaling Pathway in a Rat Model of Intracerebral Hemorrhage

**DOI:** 10.3389/fnmol.2018.00176

**Published:** 2018-05-29

**Authors:** Weilin Xu, Liansheng Gao, Tao Li, Jingwei Zheng, Anwen Shao, Jianmin Zhang

**Affiliations:** ^1^Department of Neurosurgery, Second Affiliated Hospital, School of Medicine, Zhejiang University, Hangzhou, China; ^2^Brain Research Institute, Zhejiang University, Hangzhou, China; ^3^Collaborative Innovation Center for Brain Science, Zhejiang University, Hangzhou, China

**Keywords:** mesencephalic astrocyte-derived neurotrophic factor (MANF), intracerebral hemorrhage, neuronal apoptosis, secondary brain injury, neuroprotection

## Abstract

Neuronal apoptosis plays key roles in secondary brain injury caused by intracerebral hemorrhage (ICH). This study first reported the role of mesencephalic astrocyte-derived neurotrophic factor (MANF) in alleviating secondary brain injury through anti-apoptosis in rat model of ICH. The recombinant human-MANF (rh-MANF) and selective Akt inhibitor MK2206 was administrated intracerebroventricularly 1 h after ICH. Brain water content, behavioral assessment, BBB (blood brain barrier) leakage was evaluated 24 h after the induction of ICH. Western blot analysis was used to evaluate the expression level of target proteins (MANF, mouse 3T3 cell double-minute 2 (MDM2), P53, Akt, Bcl-2, Bax, and caspase-3). Terminal deoxynucleotidyl transferase (TdT)-mediated dUTP nick end labeling (TUNEL) was applied to evaluate the neuronal cell death. Besides, whether MANF was expressed in neurons was verified with double immunofluorescence staining. The results suggested that the level of MANF, and its downstream proteins, Akt, MDM2 was upregulated and reached peak at 24 h after ICH. MANF was mainly expressed in neurons. The administration of rh-MANF could significantly increase the level of p-Akt, p-MDM2, Bcl/Bax ratio, but reduce the expression of p53, caspase-3 and neuronal death, thus ameliorate the neurological functions at 24 h after ICH. However, these effects of rh-MANF could be obviously reversed by MK2206. MANF could exert its neuronal anti-apoptotic effects via Akt/MDM2/P53 pathways. Therefore, MANF could be a valuable drug target in the treatment of ICH.

## Introduction

Intracerebral hemorrhage, which accounts for 10–15% of all stroke cases, is one of the most prevalent subtype of stroke worldwide (Feigin et al., 2009; Steiner et al., 2014). It was characterized by high mortality and mobility, especially in developing countries, which poses great burden on society (van Asch et al., 2010; Krishnamurthi et al., 2014). ICH is mainly caused by ruptured small arterioles that are degenerated caused by long-lasting hypertension (Rodríguez et al., 2017). It is a complicated process with varied pathophysiological mechanisms. The poor outcome after ICH is mainly caused by direct damages from blood accumulation and secondary injuries, such as brain edema, BBB disruption, inflammation and neuronal apoptosis (Chen et al., 2017; Delcourt et al., 2017; Wu et al., 2017). Surgical procedures have restricted indications and cover only a part of small clinical-relevant survival advantages (Pías-Peleteiro et al., 2017). Although numerous studies had focused on pharmacological treatments of ICH, no regimen with specific efficacy has been launched (Han et al., 2017; Wang et al., 2017).

Mesencephalic astrocyte-derived neurotrophic factor, one type of novel NTF family (Glembotski et al., 2012), has been reported to display cytoprotective effects in myocardial infarction and neurological diseases, such as Parkinson’s disease or ischemic stroke. The mechanisms involve anti-inflammatory, anti-oxidant, anti-apoptotic properties and ER stress prevention (Lindholm et al., 2007; Voutilainen et al., 2009; Hellman et al., 2011). The elevated level of MANF could partly due to ER stress and UPR as ER stress is also the key process in various diseases (Zhou et al., 2015; Goswami et al., 2016). MANF protein has two domains: The saposin-like domain at N-terminal could attach to lipid bilayer, and the unfolded C-terminal domain may be relative to the protection of cells from endoplasmic reticulum stress. However, the role of MANF in ICH has not been explored yet. Based on its characteristics mentioned above, we hypothesized that MANF could exert its neuroprotective roles in ICH.

Despite the fact that MANF plays multiple roles in the central nervous system, the underlying mechanisms have not been fully understood. One of the possible mechanisms might be through the activation of Akt (Hao et al., 2017). Akt, a serine/threonine kinase, exerts great effects in the regulation of cell development, growth, and survival (Paraskevopoulou and Tsichlis, 2017). The activation of Akt could phosphorylate diverse downstream factors, including MDM2, p53. MDM2 comprises several conserved domains, which provide the structural basis for its functions. The N-terminal domain could bind to tumor suppressor protein p53 and inhibit the transcriptional activity of p53. MDM-2 could bind to p53 and make it ubiquitination for proteasomal degradation. Ubiquitination activity of MDM-2 can be enhanced when MDM-2 is phosphorylated by Akt at Ser-166/186 (Zhou et al., 2001), which promotes it transfer to nuclear and interact with transcriptional co-activator p300, then exacerbate p53 degradation and inhibits p53 function (Grossman et al., 1998). On the other hand, abnormally elevated p53 activity could also trigger over express of MDM-2, which conversely suppresses p53 activation so form a feedback loop (Toth et al., 2006). Thus, we hypothesized that MANF could prevent neuronal cells from apoptosis via Akt/MDM2/p53 pathway in ICH.

In this study, we found that MANF could display its neuroprotective effects in rats after ICH via alleviating brain edema, BBB protection and neuronal apoptosis prevention. The possible underlying mechanisms may involve the activation of Akt and MDM2 and the degradation of p53, thus up-regulating the expression of anti-apoptotic proteins and down-regulating the expression of pro-apoptotic proteins.

## Materials and Methods

### Animals

All experimental protocols were warranted by the ethics committee of Zhejiang University. The procedures were conducted according to NIH guidelines. Two hundred and fourteen male SD rats (280–330 g), purchased from SLAC Laboratory Animal Co., Ltd. (Shanghai, China), were applied to this study. All the rats were kept in a 12 h day/night cycle (22 ± 1°C; 60 ± 5% humidity). Food and water were *ad libitum*.

### ICH Rat Models

**FIGURE 1:**
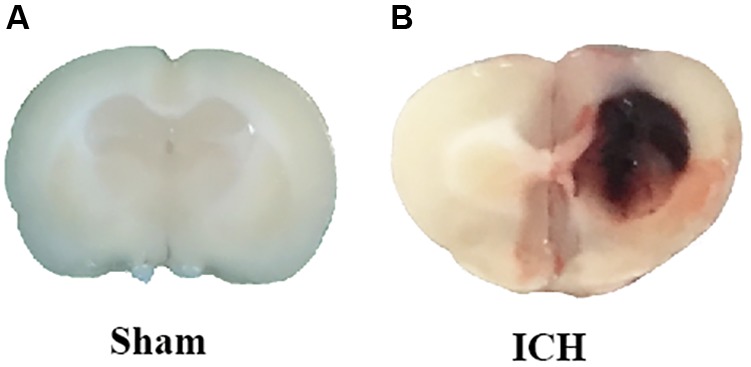
Representative pictures of brain slices in sham **(A)** and ICH **(B)** group (24 h).

The ICH models were made according to previous studies (Xie et al., 2014; Zhou et al., 2014). Deep anesthesia was applied to the rats using pentobarbital (40 mg/kg, intraperitoneal injection). The operation was performed with the aid of a stereotaxic frame (Stoelting Co., United States). First, we isolated the right femoral artery and inserted with a polyethylene catheter (PE-160) to obtain blood for the following injection. Second, the skin on the top of the head was longitudinally incised with a scalpel. Third, we drilled a burr hole at the place 3.5 mm lateral right of the bregma. One hundred microliter autologous blood, obtained from the right femoral artery, was manually injected into the right striatum (5.5 mm depth) using a Hamilton syringe with a 26 G needle. After the injection, the needle was kept in place for additional 10 min. Finally, we blocked the burr hole with a sterilized medical bone wax and closed the incision with sutures. The rats in sham-group received the same procedures except for the insertion of the needle (**Figure 1**).

### Experimental Design

In first step, the time course of MANF, p-Akt and p-MDM2 was determined after the induction of ICH. Seventy-four rats were randomly distributed to seven groups: sham (*n* = 12), 3 h (*n* = 10), 6 h (*n* = 10), 12 h (*n* = 10), 24 h (*n* = 12), 48 h (*n* = 10), and 72 h (*n* = 10). Six brains per group were sampled for Western blot analysis. Immunofluorescence staining of MANF with neuronal nuclei (NeuN) was conducted in sham (*n* = 2) and 24 h after ICH (*n* = 2).

In second step, we explored the effects of treatment with rh-MANF, eighty rats were randomly distributed into four groups: sham (*n* = 20), ICH (*n* = 20), ICH+ vehicle (10 μl sterile saline, *n* = 20), ICH+ MANF (5 μg in 10 μl sterile saline, *n* = 20). We assessed neurological functions, brain water content and EB extravasation at 24 h after ICH in each group (*n* = 6). The expression of MANF, MDM2, P53, Bcl-2/Bax ratio and caspase-3 was analyzed by Western blot at 24 h after ICH (*n* = 6). Immunofluorescence staining of TUNEL and NeuN was also conducted in all groups at 24 h after ICH (*n* = 4).

In step three, in order to further explore the underlying mechanisms of neuroprotective effects of MANF, 16 rats were randomly distributed into four groups: sham (*n* = 15), ICH+ vehicle (*n* = 15), ICH + MANF (5 μg in 10 μl in sterile saline, *n* = 15), or ICH+ MANF (5 μg, Sino biological inc., Beijing, China) + MK2206 (100 μg, *n* = 15, Selleck Chemicals, Houston, TX, United States). rh-MANF and MK2206 was applied intracerebroventricularly at 1 h after ICH. The levels of MANF, Akt, MDM2, P53, Bcl-2/Bax ratio, and caspase-3 were evaluated at 24 h after ICH by Western blot analysis in each group (*n* = 6). Immunofluorescence staining of TUNEL and NeuN was also conducted in all groups at 24 h after ICH (*n* = 4).

### Behavioral Assessment

Neurological function was assessed at 24 h after ICH with a marking system called the NSS (Cui et al., 2017). The NSS was graded with a scale ranging from 1 to 18 (Supplementary Table 1).

### Brain Water Content

The brain water content assessment was performed at 24 h after ICH, which was based on wet–dry method. In brief, the brain hemispheres of the rats were quickly removed after anesthetization. Then, the injured brain hemisphere was weighed (wet weight). Next, the hemisphere was put in an oven for 72 h (105°C, dry weight). Finally, the brain water content was evaluated as follows: [(wet weight – dry weight)/(wet weight)] × 100% (Chen et al., 2015).

### Evans Blue Staining

Blood–brain barrier leakage was assessed via EB staining at 24 h after ICH. Two percent EB solution (8 mL/kg, Sigma–Aldrich) was applied through femoral vein after anesthetization. Two hours later, the rats received transcardial perfusion with 0.1M PBS. Next, injured brain hemisphere was collected and homogenized in N, N-dimethylformamide. The sample was incubated in water bath (50°C) for 48 h and centrifuged at 12,000 × *g* for 30 min. Finally, the supernatant was collected and measured at 620 nm with a spectrophotometer (2,000°C, Thermo Fisher) (Zhao et al., 2016).

### Immunofluorescence and Calculation of Apoptotic Cells

After anesthetization, transcardial perfusion with 0.1M PBS was performed, followed by another perfusion with 4% paraformaldehyde (pH 7.4). Then the cerebral hemispheres were removed and put into 4% PFA for post-fixation (4°C, 24 h). After that, the brains were transferred to sucrose solution (30%, 2 days). Next, the brains were coronally sliced into 10 mm sections, which were then fixed on slides and used for immunofluorescence staining, and then blocked with 10% normal goat serum for 2 h at room temperature and incubated at 4°C overnight with primary antibodies: rabbit anti-MANF (1:500, Abcam ab67271), anti-Caspase3 (1:200, Abcam ab13847), mouse anti-NeuN (1:500, Abcam ab177487). After that, secondary antibodies were applied for 2 h at room temperature. Finally, a fluorescence microscope (Olympus, Tokyo, Japan) was used to observe the sections and the photographs taken were post-processed with Photoshop 13.0 (Adobe Systems Inc., Seattle, WA, United States). In addition, we used TUNEL (Roche Inc., Basel, Switzerland) staining to quantitatively evaluate the cell apoptosis. Neuronal apoptosis was assessed by the proportion of TUNEL and Caspase-3 positive neurons in six sections at ×200 magnification of each brain sample. The results were showed as cells per square millimeter.

### Western Blot Analysis

After being anesthetized, the peri-hematoma brain tissue of the rat was collected and further processed as previously reported (Nakka et al., 2010). Forty microgram protein from each sample was used for electrophoresis (100 V, 1 h) and then transferred to the polyvinylidene fluoride membranes at 250 V for 1 h. Next, the protein was incubated overnight (4°C) with rabbit anti-MANF (1:2000, Abcam ab67271), anti-p-Akt (1:2000, CST 4060s), anti-Akt (1:1000, CST 9272s), anti-p-MDM-2 (1:1000, CST 3521s), anti- MDM-2 (1:1000, CST 86934s), anti-p53 (1:1000, CST 2527s), anti-Bax (1:1000, Abcam ab32503), anti-Bcl-2 (1:500, Abcam ab59348), anti-caspase3 (1:500, Abcam ab13847), and mouse anti-β-actin (1:5000, Abcam ab8226). After that, secondary antibodies (1:10000, Zhongshan Gold Bridge ZB-2301 or ZB-2305) were applied at room temperature for 1 h. Finally, protein was visualized with the ECL Plus chemiluminescence reagent kit (Amersham Bioscience, Arlington Heights, IL, United States). The results were showed as the relative density which was the ratio of the grayscale value of the target proteins to that of β-actin, pan-Akt, or pan-MDM2.

### Transmission Electron Microscopy

The rats received transcardial perfusion with 0.1M PBS and 4% paraformaldehyde (pH 7.4) after anesthetization. Then the perihematomal tissues were collected and grained into 1 mm^3^ slices. After that, the slices were immersed into glutaraldehyde (2.5%) at 4°C overnight. Next, we put the samples in 1% osmium tetroxide for 1 h and dehydrated the samples with a serious of graded ethanol. Then the tissues were immersed into a mixture of propylene oxide and resin (1:1). Four hours later, the samples were imbedded in resin. After that, we cut the samples in to 100 nm sections and stained the sections with 4% uranyl acetate (20 min) and 0.5% lead citrate (5 min). Finally, the transmission electron microscopy (Philiphs Tecnai 10) was used to observe the ultrastructure of brain tissues.

### Statistical Analysis

Results were presented as mean ± SD. Further analysis was performed by SPSS 22.0 software (IBM, United States). Student’s *t*-test or one-way analysis of variance was applied for the comparisons between groups, with a *p* < 0.05 deeming to be statistically significant.

## Results

### Physiological Data

Data regarding the physiological parameters were collected during surgical procedures. No significant differences of physiological parameters were observed across each group (data not shown).

### Expression Level of MANF, p-Akt and p-MDM2 After ICH

**FIGURE 2:**
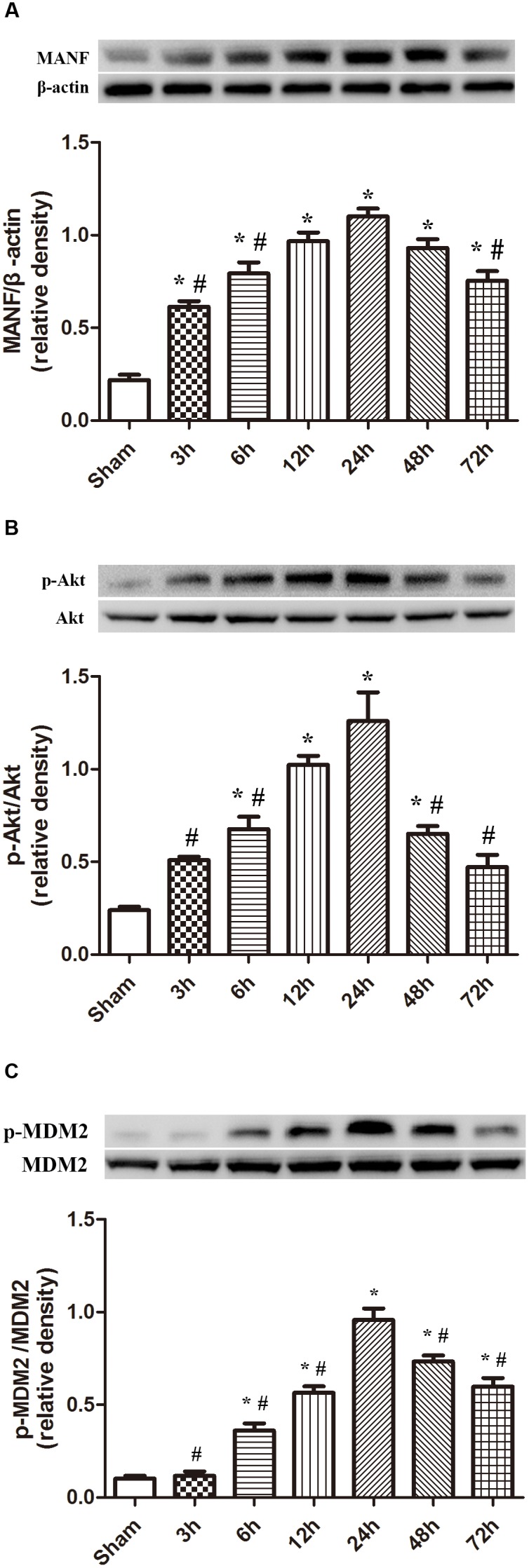
Expression of MANF, p-Akt and p-MDM2. **(A)** Time course of MANF in injured hemisphere after ICH; **(B)** Time course of p-Akt; **(C)** Time course of p-MDM2; *n* = 6 for each group. The bars represent the mean ± SD. ^∗^*p* < 0.05 vs. sham, #*p* < 0.05 vs. ICH at 24 h.

The protein level of MANF started to raise at 3 h, and peaked at 24 h after ICH (*p* < 0.05, **Figure 2**). While the level of p-Akt and p-MDM2 increased at 6h, and peaked at 24 h after ICH (*p* < 0.05, **Figure 2**).

### Morphometric Changes of Brain Tissues 24 h After the Induction of ICH

**FIGURE 3:**
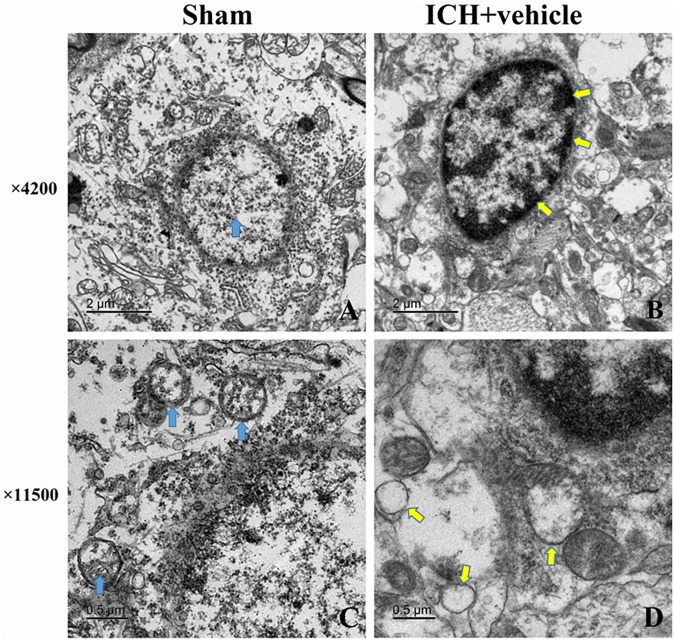
Transmission electron microscopy images of the morphometric changes of brain tissues for sham group and ICH + vehicle group. **(A)** Sham, the arrow (green) indicated normal nucleus with homogeneous chromatin; **(B)** ICH+ vehicle (scar bar = 2 μm), the arrows (white) indicated abnormal nucleus with condensed chromatin; **(C,D)** Magnification of **(A,B)** (scar bar = 0.5 μm), the arrows(green) in **(C)** indicated normal mitochondria with intact prominent cristae, while arrows (white) in **(D)** indicated vacuole and swollen mitochondria.

We observed the morphometric changes of mitochondria and nucleus under the help of TEM. In the sham group, prominent cristae within mitochondria and intact membrane structure could be observed (**Figures 3A,C**). Chromatin was homogeneous distributed within the nucleus and large oval nucleus with clear nuclear membrane was observed for normal nucleus. In the ICH group, the heterogeneities of mitochondria and chromatin were obvious. neurons had irregular nuclear membrane, chromatin condensation, many vacuole and swollen mitochondria (**Figures 3B,D**).

### MANF Distribution in Cells After ICH

**FIGURE 4:**
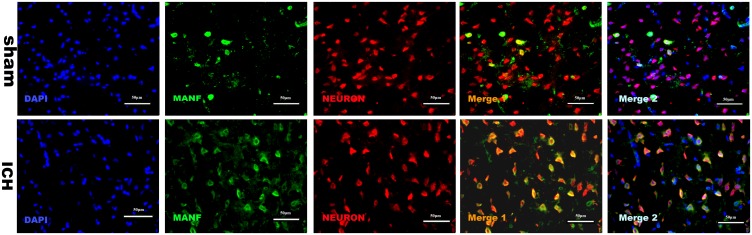
Representative microphotographs of immunofluorescence staining showing localization of MANF (*green*) and NeuN (*red*) in the perihematomal region after ICH. MANF was upregulated after the induction of ICH and mainly located in neurons. *N* = 2 for each group. Scale bar = 50 μm.

The results of double immunofluorescence staining of MANF with NeuN in both sham and ICH groups (24 h) showed that MANF was mainly located in neurons (**Figure 4**) and the protein level of MANF increased 24 h after ICH.

### Brain Edema, BBB Permeability and Neurological Functions at 24 h After ICH

**FIGURE 5:**
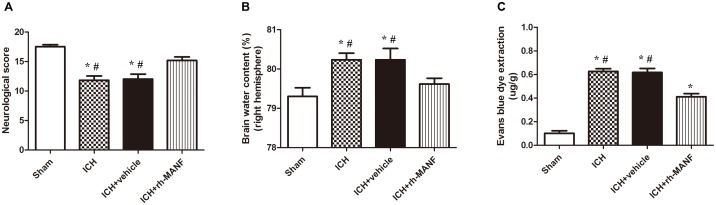
Effects of rh-MANF on neurological functions, brain edema and BBB leakage. **(A)** The quantification of neurological functions; **(B)** The quantification of brain water content at 24 after ICH; **(C)** The quantification of Evans blue dye extravasation at 24 after ICH. *n* = 6 for each group. The bars represent the mean ± SD. ^∗^*p* < 0.05 vs. sham, #*p* < 0.05 vs. ICH at 24 h.

A dose of 5 μg recombinant human MANF (rh-MANF) was administered intracerebroventricularly 1 h after the induction of ICH. Brain water content and BBB permeability and neurological functions were measured at 24 h after ICH. The induction of ICH could significantly increase the brain water when compared with the rats in sham group (*p* < 0.05, **Figure 5B**). However, the administration of rh-MANF could obviously reduce the water content at 24 h after ICH (*p* < 0.05 vs. ICH+ vehicle, *n* = 6, **Figure 5B**). Besides, the RB was obviously increased in ipsilateral hemisphere of ICH compared with the rats in sham group (*p* < 0.05 vs. sham, *n* = 6, **Figure 5C**), while the rats receiving rh-MANF displayed reduced levels of EB staining compared with the rats in ICH+ vehicle group (*p* < 0.05 vs. sham, *n* = 6, **Figure 5C**). Severe neurological deficits were observed in the ICH group when compared with the sham group at 24 h after ICH (*p* < 0.05, *n* = 6, **Figure 5A**). However, the administration of rh-MANF could significantly improve the neurological functions (*p* < 0.05 vs. ICH+ vehicle, *n* = 6, **Figure 5A**).

### Administration of rh-MANF Promotes Neuronal Survival at 24 h After ICH

**FIGURE 6:**
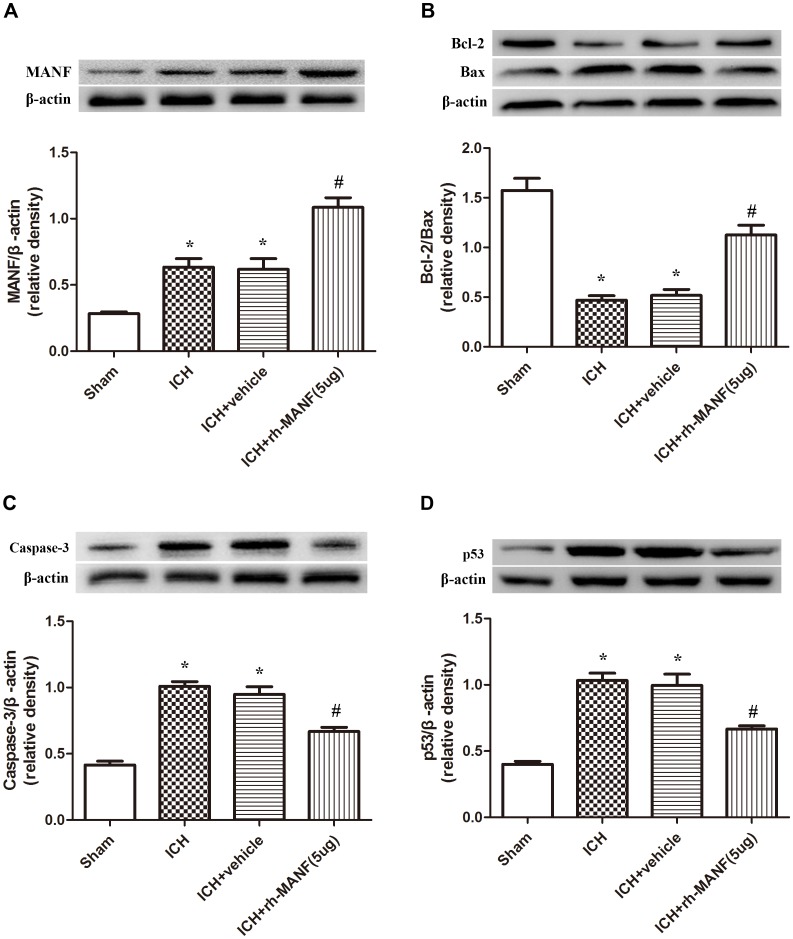
The administration of rh-MANF increased the expression of MANF and bcl-2, but decrease the level of Bax, Caspase-3 and p53. **(A)** MANF; **(B)** Bcl-2, Bax; **(C)** Caspase-3; **(D)** p53. *n* = 6 for each group. The bars represent the mean ± SD. ^∗^*p* < 0.05 vs. sham, #*p* < 0.05 vs. ICH+ vehicle.

**FIGURE 7:**
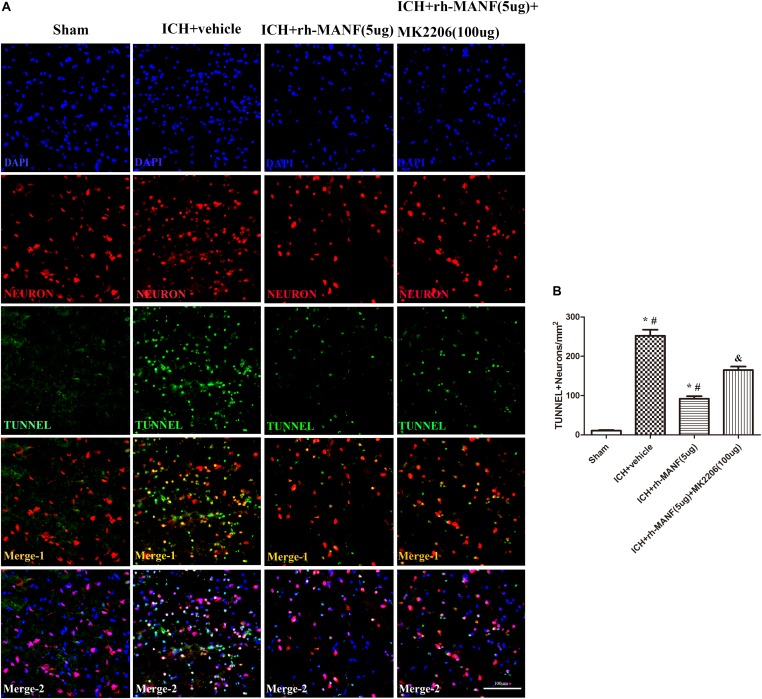
The administration of rh-MANF significantly decreased the number of TUNEL and NeuN double-stained cells in the perihematomal region 24 h after ICH, which could be obviously reversed by MK2206 (100 μg). **(A)** Representative microphotographs showed the co-localization of NeuN (*red*) with TUNNEL (*green*)-positive cells in injured brain hemisphere at 24 h after ICH; **(B)** Quantitative analysis of TUNNEL-positive neurons showed that rh-MANF decreased the number of apoptotic cells after ICH. *Scale bar* = 100 μm. ^∗^*p* < 0.05 vs. sham, #*p* < 0.05 vs. ICH+ vehicle; &*p* < 0.05 vs. ICH+ rh-MANF.

**FIGURE 8:**
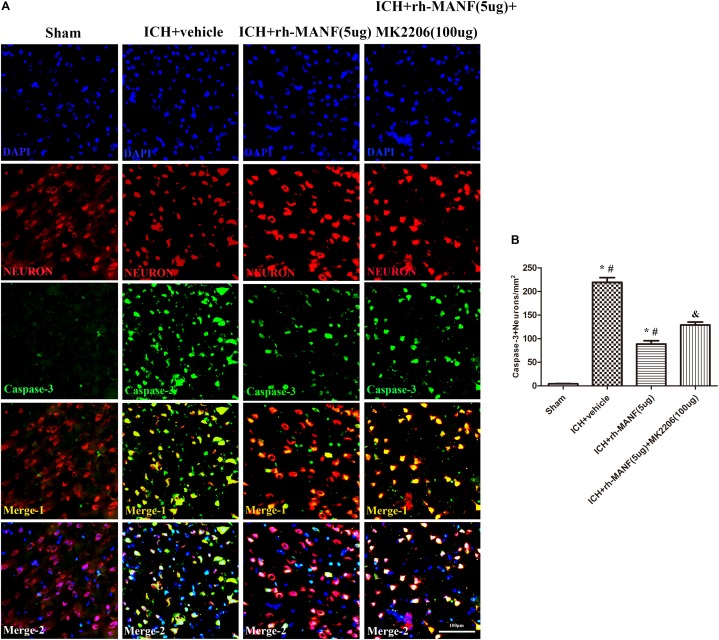
The administration of rh-MANF significantly decreased the number of Caspase-3 and NeuN double-stained cells in the perihematomal region 24 h after ICH, which could be obviously reversed by MK2206 (100 μg). **(A)** Representative microphotographs showed the co-localization of NeuN (red) with Caspase-3 (green)-positive cells in injured brain hemisphere at 24 h after ICH; **(B)** Quantitative analysis of Caspase-3 positive neurons showed that rh-MANF decreased the number of apoptotic cells after ICH. *Scale bar* = 100 μm. ^∗^*p* < 0.05 vs. sham, #*p* < 0.05 vs. ICH+ vehicle; &*p* < 0.05 vs. ICH+ rh-MANF.

The protein level of MANF was significantly elevated at 24 h after ICH. However, the administration of rh-MANF could increase the total amount of MANF, which could further up-regulate the expression of p-MDM2 but reduce the expression level of p53 compared with ICH+ vehicle group (*p* < 0.05) (**Figures 6A,D**). Additionally, the induction of ICH significantly decreased the ratio of Bcl-2/Bax (**Figure 6B**) while upregulated the level of caspase-3 (*p* < 0.05, ICH+ vehicle vs. sham groups) (**Figure 6C**). However, administration of rh-MANF could significantly reversed these results (*p* < 0.05 ICH+ rh-MANF vs. ICH+ vehicle). The results of TUNEL staining suggested that the number of TUNEL and NeuN double-stained cells significantly increased at 24 h after ICH, as well as Caspase-3 (*p* < 0.05, ICH vs. sham, **Figures 7**, **8**). Whereas the number of TUNEL-positive neurons and Caspase-3 positive neurons were significantly decreased after the administration of rh-MANF (*p* < 0.05, ICH+ rh-MANF vs. ICH+ vehicle).

### Role of Downstream Akt in the MANF-Mediated Neuroprotective Effects 24 h After ICH

**FIGURE 9:**
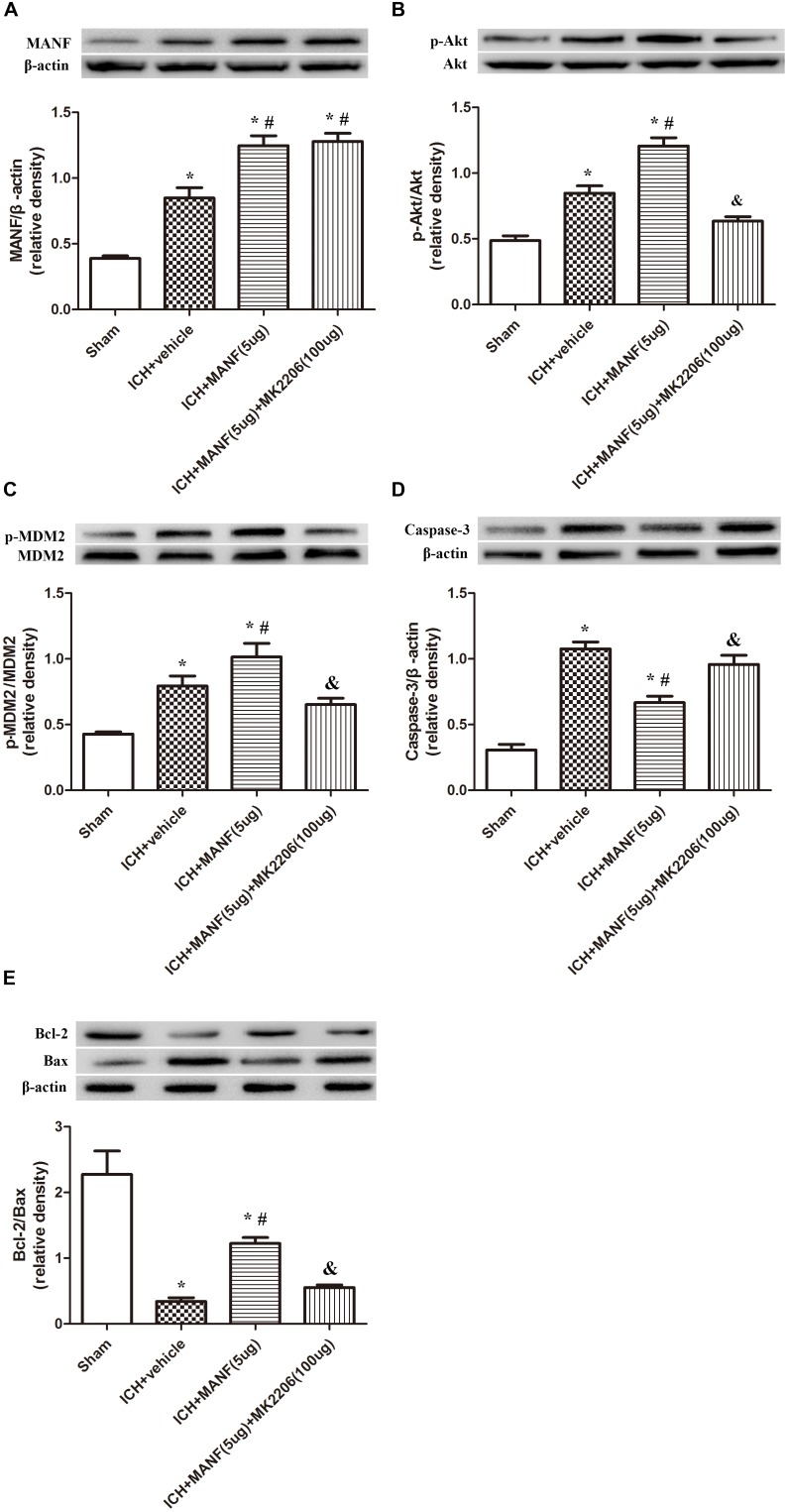
The administration of rh-MANF significantly decreased the number of Caspase-3 and NeuN double-stained cells at 24 h after ICH, which could be obviously reversed by MK2206 (100 μg). **(A)** MANF; **(B)** p-Akt; **(C)** p-MDM2; **(D)** Caspase-3; **(E)** Bcl-2 and Bax. *n* = 6 for each group. The bars represent the mean ± SD. ^∗^*p* < 0.05 vs. sham, #*p* < 0.05 vs. ICH+ vehicle, &*p* < 0.05 vs. ICH+ rh-MANF.

In order to explore the effects of Akt in the MANF-mediated neuroprotective effects, MK 2206, a highly selective inhibitor of Akt, was applied at 1 h after ICH. The results showed that the expression of MANF, which was significantly increased at 24 h after ICH, was not obviously affected by the administration of MK2206 (**Figure 9A**). However, upregulation effects of p-Akt induced by administration of rh-MANF was significantly suppressed by MK 2206 (*p* < 0.05 vs. ICH+ rh-MANF, **Figure 9B**). Besides, the administration of rh-MANF could greatly enhanced cell survival via increasing Bcl-2/Bax ratio while decreasing the level of caspase-3 (*p* < 0.05 vs. ICH+ vehicle, **Figures 9C–E**); However, this neuroprotective effects could be significantly weakened by MK 2206 (*p* < 0.05 vs. ICH+ rh-MANF).

## Discussion

In this study, we explored the role of MANF in rats after the induction of ICH. The expression of MANF was noted to be up-regulated after ICH insult, and the downstream target proteins of MANF including Akt and MDM2, reached peak at 24 h after ICH. Besides, the expression level of p53 was significantly upregulated after ICH. MANF was expressed mainly in neurons. The result of administration of rh-MANF suggested that MANF could exert neuroprotective effects in rats after experimental ICH. rh-MANF could significantly alleviate the neurological deficits, reduce brain edema, protect BBB and prevent neuronal apoptosis by increasing Akt phosphorylation, and Bcl-2/Bax ratio, reducing the level of caspase-3. However, the anti-apoptotic effects of rh-MANF could be greatly weakened by the administration of selective Akt inhibitor – the MK 2206.

In addition, we also used TEM to document the morphometric changes of the ultrastructure of mitochondria and nucleus after ICH. As a result, irregular nuclear membrane, chromatin condensation, many vacuole and swollen mitochondria was observed in the ICH group, which was the classical manifestation of cellular apoptosis. Besides, it was reported that the protein level of MANF was highly elevated in traumatic brain injury and ischemic stroke (Yang et al., 2014; Chiu et al., 2016). The MANF could protect neurons from apoptosis via activating the PI3K/Akt pathway (Hao et al., 2017). In addition, MANF is an ER stress-inducible protein (Lin et al., 1993; Mizobuchi et al., 2007). The special structure of MANF determined it has a unique mechanism to rescue neurons from death (Hellman et al., 2011). In this study, we explored the expression level of MANF and its downstream targets, p-MDM2 and P53 after ICH. In accordance with the abovementioned observations, the protein level of MANF was obviously upregulated at 3 h while the level of p-Akt and p-MDM2 start to increase at 6 h, all of which reached the highest at 24 h. The expression of P53 was down-regulated, which reached its nadir at 24 h. According to the abovementioned, MANF displayed a close relationship with the elevated pro-survival signals in experimental ICH models, which was consistent with previous reported studies that the level of MANF was up-regulated in neurological diseases (Stratoulias and Heino, 2015; Norisada et al., 2016).

We further investigated the effects of MANF by the intracerebroventricular injection of rh-MANF at 1 h after ICH. Intracranially (extracellularly) injected MANF effectively protected dopaminergic neurons in a rat model of 6-hydroxydopamine induced Parkinson’s disease (Voutilainen et al., 2009) and the ischemia model (Mikko et al., 2009), thereby suggesting it to function as a secreted neurotrophic factor (Lindholm et al., 2007; Mikko et al., 2009; Voutilainen et al., 2009). It may function on neurons through activating a transmembrane protein receptor and induce intracellular second messengers. In this study, the administration of rh-MANF could improve neurobehavioral deficits, alleviate BBB disruption and reduce brain edema at 24 h after ICH. Besides, this treatment could significantly increase in the expression of MDM2 and reduce the expression of P53. In addition, the expression of Bcl-2 was upregulated and that of Bax as well as caspase-3 was down-regulated. Double immunofluorescence staining demonstrated that neuronal apoptosis was induced by ICH, while the administration of rh-MANF significantly promoted neuronal survival. These results demonstrated that MANF exerted neuroprotective effects in experimental ICH models. This was consistent with the characteristics of MANF in previously reported studies. Airavaara et al. (2009) treated the cerebral cortex with recombinant human MANF 60-min before the middle cerebral artery occlusion (MCAO), the results demonstrated that MANF exerts its neuroprotective role in cerebral ischemia via preventing the cells from necrosis/apoptosis in cerebral cortex. *In vivo*, Sun et al. (2017) found that MANF could reduce cell death by increasing the level of HSP70 in SHSY-5Y cells. In addition, ER stress which played great roles in ICH could also be regulated by MANF in ischemia/reperfusion models. The use of rh-MANF after cerebral ischemia could significantly decrease the ischemic volume and reduce cerebral injury by regulating ER stress and UPR (Voutilainen et al., 2015).

**FIGURE 10:**
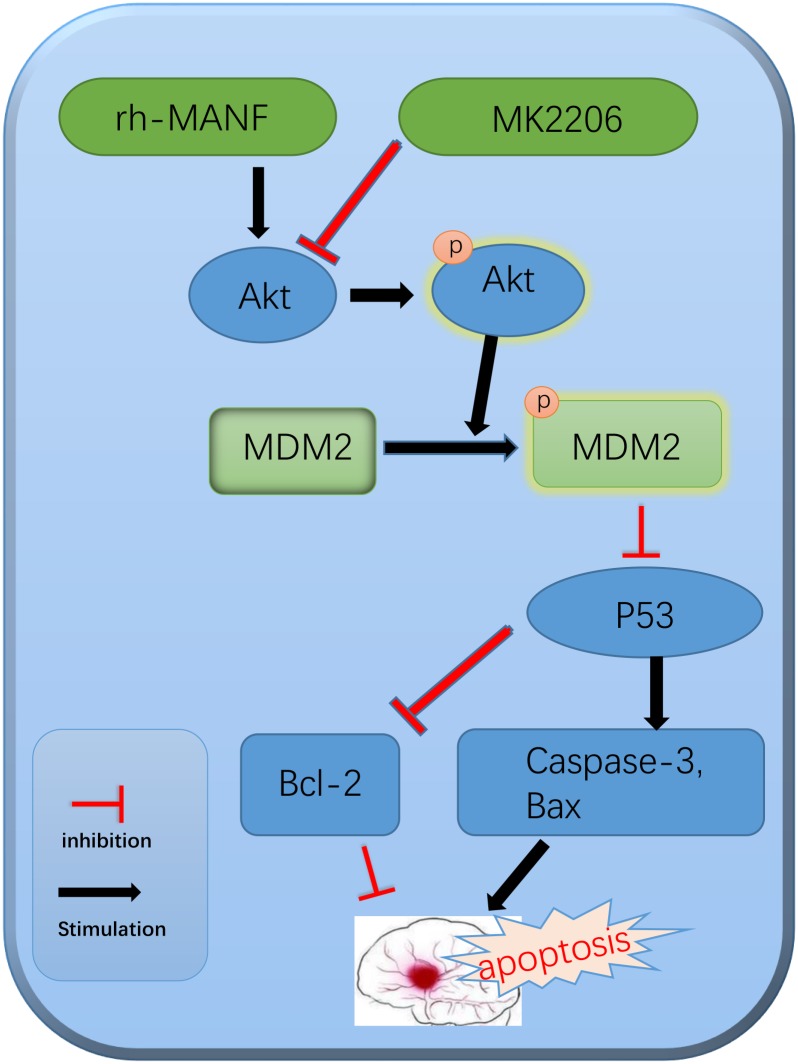
The potential molecular mechanisms of MANF-mediated anti-apoptotic effects via Akt/MDM2/p53 pathway.

In addition, we explored the role of Akt in MANF-mediated neuroprotection. Many researchers have demonstrated that Akt signaling was critical in the promotion of neuronal survival either in physiological or pathological condition (Zhao et al., 2014; Song et al., 2015). In this study, the results suggested that the level of p-Akt significantly increased at 6 h and peaked at 24 h after ICH. The administration of rh-MANF could further increase the level of p-Akt, as well as p-MDM2, Bcl-2 while reduce the expression of p53, Bax and caspase-3. However, the results could be obviously reversed by the use of Akt inhibitor MK2206. The results demonstrated that MK2206 could partly counteract the neuroprotective effects of rh-MANF. All the above-mentioned results proved the potential value of rh-MANF in the treatment of ICH via Akt/MDM2/P53 pathway (**Figure 10**).

Although this study verified the neuroprotection effects of MANF, some limitations could not be ignored. Firstly, MANF has been reported to exert its neuroprotective effects in many ways (Lindahl et al., 2017). This study only focused on its anti-apoptotic characteristics without further investigation of its role in anti-inflammation or autophagy. Secondly, the anti-apoptotic pathway of MANF in this study was limited to Akt/MDM2/p53. However, some other signal pathways were also reported in neurological diseases (Lindholm and Saarma, 2010). Hence further studies on the relationship of MANF and other signal pathways in neuronal apoptosis after ICH are also required.

## Author Contributions

WX, LG, and TL contributed to the conception, design, and drafting of the work. WX and JZ performed the data analysis. LG and JZ made animal models and collected the samples. TL and WX performed the molecular biology experiment. WX and LG performed the immunofluorescence staining. AS and JZ revised the manuscript critically for important intellectual content. The study was completed with contributions from all authors. All authors have given approval to the final version of the manuscript.

## Conflict of Interest Statement

The authors declare that the research was conducted in the absence of any commercial or financial relationships that could be construed as a potential conflict of interest.

## Abbreviations

BBB: blood brain barrier
EB: Evans blue
ICH: intracerebral hemorrhage
MANF: mesencephalic astrocyte-derived neurotrophic factor
MDM2: mouse 3T3 cell double-minute 2
NSS: Neurological Severity Score
NTF: neurotrophic factor (NTF)
SD: Sprague–Dawley
TUNNEL: Terminal deoxynucleotidyl transferase (TdT)-mediated dUTP nick end labeling
UPR: unfolded protein response
