# Supplementary material for: Mesencephalic Astrocyte-Derived Neurotrophic Factor (MANF) Protects Against Neuronal Apoptosis via Activation of Akt/MDM2/p53 Signaling Pathway in a Rat Model of Intracerebral Hemorrhage
Source: Front Mol Neurosci. 2018 May 29;11:176. doi: 10.3389/fnmol.2018.00176 (PMC5987019; doi:10.3389/fnmol.2018.00176)
Supplement: Supplementary file 1 [file Table_1.DOCX]

| **Supplemental TableⅠ. Neurological Severity Scores (NSS)** | | |
| --- | --- | --- |
|  | **Items** | **Score** |
| **raising rat by tail (normal=0; maximum=3)** | | (3) |
|  | flexion of forelimb | 1 |
|  | flexion of hindlimb | 1 |
|  | head moved >10° to vertical axis within 30s | 1 |
|  | placing rat on floor (normal; maximum=3) | (3) |
|  | normal walk | 0 |
|  | inability to walk straight | 1 |
|  | circling toward paretic side | 2 |
|  | falls down to paretic side | 3 |
| **sensory tests (normal=0; maximum=2)** | | (2) |
|  | placing test (visual and tactile test) | 1 |
|  | proprioceptive test (deep sensation) | 1 |
| **beam balance tests (normal=0; maximum=6)** | | (6) |
|  | balances with steady posture | 0 |
|  | grasps side of beam | 1 |
|  | hugs beam and 1 limb falls down from beam | 2 |
|  | hugs beam and 2 limbs falls down from beam, or spins on beam(>60s) | 3 |
|  | attempts to balance on beam but falls off(>40s) | 4 |
|  | attempts to balance on beam but falls off(>60s) | 5 |
|  | falls off; no attempt to balance or hang on to beam(<20s) | 6 |
| **reflex absence and abnormal movements** | | (4) |
|  | pinna reflex (head shake when auditory meatus is touched) | 1 |
|  | corneal reflex (eye blink when cornea is lightly touched with cotton) | 1 |
|  | startle reflex (motor response to a brief noise) | 1 |
|  | seizure, myoclonus, myodystony | 1 |
| **maximum points** | | (18) |
